# Lived experiences of missing with dementia and pathways to care: a qualitative study of carers’ and professionals’ perspectives

**DOI:** 10.1093/ageing/afaf289

**Published:** 2025-10-10

**Authors:** Phuong Leung, Valerie Lye, Hoi Tat Kwok, Lawrence Fong, Vasiliki Orgeta

**Affiliations:** Division of Psychiatry, Faculty of Brain Sciences, UCL, London, England, UK; Division of Psychiatry, Faculty of Brain Sciences, UCL, London, England, UK; Division of Psychiatry, Faculty of Brain Sciences, UCL, London, England, UK; Division of Psychiatry, Faculty of Brain Sciences, UCL, London, England, UK; Division of Psychiatry, Faculty of Brain Sciences, UCL, London, England, UK

**Keywords:** missing incidents, dementia, carers, professionals, care pathways, qualitative research, older people

## Abstract

**Background:**

People with dementia are at increased risk of going missing, which can lead to severe harm and distress. Despite the need for a better understanding of how to support people with dementia and their families affected, research in this area remains limited. This qualitative study explored lived experiences of families affected by missing incidents, and carers’ and professionals’ perspectives of how to improve care.

**Methods:**

A multi-perspective, qualitative framework analysis approach was undertaken with purposive sampling. Topic guides were developed in consultation with carers and professionals. We conducted in-depth semi-structured interviews and a focus group with key stakeholders (*n* = 33), which included carers of people with dementia (*n* = 9), police officers (*n* = 6), healthcare professionals (*n* = 12) and researchers (*n* = 6). Data were interpreted using thematic analysis to identify key themes. Reflexivity was considered throughout the process.

**Results:**

Five key themes were identified: (i) risk and protective factors of missing incidents, (ii) gaps in reporting and response pathways, (iii) the use of technology for preventing and responding to missing incidents, (iv) the role of communities in prevention of harm, and (v) the need for integration of services through multi-agency collaboration.

**Conclusion:**

This study highlights key gaps on our national and global response to missing incidents in dementia. Understanding risk and protective factors and improving our public health response through multi-agency collaboration and clear care pathways, were considered key by stakeholders. Our findings offer actionable insights to inform future strategies and improve care for people with dementia who go missing.

## Key Points

- Missing incidents in dementia are associated with significant harm and distress for people with dementia and their families.
- Multiple risk factors increase the risk of experiencing a missing incident.
- Our response to missing incidents in dementia remains fragmented with unclear care pathways.
- Innovative technology, and psychoeducation initiatives are urgently needed to protect people with dementia from harm and distress associated with missing incidents.
- Standardisation of reporting, and multi-agency collaboration are key in improving our public health response to dementia-related missing incidents.

## Introduction

Missing incidents involving people with dementia represent a significant public health challenge worldwide, affecting not only individuals with dementia but also their families and local communities [1]. Experiencing a missing incident is associated with increased risk of severe harm and distress for both people with dementia and their carers [2, 3]. Research suggests that up to 70% of people living with dementia will experience at least one missing incident during the course of the condition [4], with those remaining missing for more than 24 hours facing an increased risk of serious injury or death [2, 5]. While evidence on the harms associated with missing incidents continues to grow, comparatively little research has examined the factors that exacerbate these harms, and understanding the lived experiences of those affected [6].

Evidence from multiple countries demonstrates the severe and often life-threatening consequences of dementia-related missing incidents [7]. In Canada, such incidents have been linked to unanticipated injuries, institutionalisation, and death [8], while in Sweden, delayed reporting and prolonged search efforts were found to increase the risk of adverse outcomes [9]. In Japan, common harms included trauma from falls or traffic accidents, dehydration and hypothermia [2], whereas in the UK, older age, longer duration missing and delays in reporting were identified as key risk factors for severe harm, including death [3]. Collectively, these findings highlight the profound impact of missing incidents and reinforce the urgent need for targeted prevention strategies to protect people with dementia and reduce the likelihood of harm [10].

With the projected rise in the number of people living with dementia [11], missing incidents are anticipated to remain a significant challenge for families, health and social care services, and the police, who are frequently the first responders [8, 12]. Gaining insight into the lived experiences of families [6, 13], and understanding how different stakeholders respond are essential for the development of effective interventions [12, 14]. This need is particularly salient given that dementia care and support for families remain fragmented, pathways for preventing missing incidents are underdeveloped [7, 15], and only a limited number of interventions currently exist to reduce or mitigate the risks associated with these events [16].

A key barrier to improving care is the limited understanding of lived experiences [17], and of how existing systems operate in practice [18], challenges that are further compounded by the constraints faced by carers and overstretched social care services [19]. Investigating the perspectives of families, alongside professionals and first-responders, can provide critical insights for the design of sustainable interventions that are both feasible and responsive to the needs of those most affected [20, 21].

This study was designed to address this important gap by enhancing understanding of lived experiences and examining the systems currently in place to safeguard people with dementia who go missing. To our knowledge, this is the first study to explore both the lived experience of families, and the perspectives of carers and professionals on how care can be improved. The research questions addressed were: (i) What are the lived experiences of families who have experienced a missing incident? (ii) What systems, mechanisms, and pathways currently exist to support people with dementia who go missing? and (iii) What interventions and strategies are needed to mitigate risk and prevent harm?

## Methods

### Sample recruitment

Family carers were recruited through the Join Dementia Research database https://www.joindementiaresearch.nihr.ac.uk/, whereas police officers and healthcare professionals were recruited via media outlets, professional networks or third sector organisations such as Age UK and Missing People. Participants received detailed information about the study, and a voucher for their participation. Written consent was obtained from all participants before their scheduled interview or focus group. Inclusion criteria for participants were the experience of a missing episode (carers) or being directly involved in the care of people with dementia who experience a missing episode (professionals). Researchers and academics were included based on their expertise and insights into missing incidents, and dementia care systems, and pathways. Participants received a £20 voucher as reimbursement for their time. Ethical approval was received from the UCL Ethics Committee, Project ID: 26113/002. Given the sensitive nature of missing incidents in dementia, participants were approached with care, and measures were in place to support emotional wellbeing, including the option to pause or withdraw from discussions, with participants offered information on relevant support services if needed.

### Data collection procedures

Data were collected through semi-structured, in-depth interviews with family carers and professionals via Zoom and/or Microsoft Teams, each lasting ~45 minutes. Data for the stakeholders’ focus group were collected in person and via Microsoft Teams during a 1 hour and 30-minute meeting. All interviews were transcribed verbatim, with data collected between October 2023 to July 2024.

Topic guides were developed after a review of key studies and a consultation with UCL’s Missing with dementia Patient and Public Involvement group. The interviews explored the circumstances surrounding people with dementia going missing, potential triggers, associated risk of harm and factors that could help prevent future incidents. Additional topics included types of support available, reporting and response pathways of missing incidents, with a focus on effectiveness and safeguarding.

Participants were provided with the option to engage in either an interview or a focus group, or both, with four individuals selecting to participate in both the interview and the stakeholders’ focus group. Both methods were guided by similar topic frameworks to ensure consistency in the issues explored, while the stakeholder group additionally facilitated interactive discussion and the generation of shared perspectives.

### Data analysis

All qualitative data were independently analysed by two researchers using framework analysis [22]. This involved: (i) familiarisation with each interview and considering important contextual notes, (ii) coding, by applying a paraphrase to classify the data, (iii) developing a working analytical framework by grouping codes into categories, (iv) applying the analytical framework by indexing transcripts using the existing codes, and (v) charting by summarising data into categories derived from each transcript. Inductive coding was used to ensure important aspects of the data were not missed.

## Results

Nine family carers, six police officers, twelve healthcare professionals and six researchers participated in the study. Demographic characteristics of the sample are presented in Table 1. The stakeholder focus group consisted of family carers, police officers, healthcare professionals, and researchers.

**Table 1:** Participant demographics.

	All participants *n* = 33
Carers *n* = 9	M (SD) or %
Age	60.22 (13.5)
Female	6 (66.7)
Ethnicity	
White British/Irish	7 (77.8)
Asian/Asian British	1 (11.1)
Other	1 (11.1)
Relationship to person with dementia	
Spouse/partner	3 (33.3)
Child/Child in law	5 (55.6)
Other	1 (11.1)
Caring more than 2 years	9 (100)
People with dementia	
Age	79.78 (9.40)
Female	5 (55.6)
Dementia type	
Alzheimer’s disease	6 (66.7)
Vascular dementia	2 (22.2)
Dementia with Lewy bodies	1 (11.1)
Living status at time of interview	
Own home	5 (55.6)
Nursing home	4 (44.4)
Professionals *n* = 24	
Age	
25–44	8 (33.4)
45–64	16 (66.6)
Female	14 (58.3)
Ethnicity	
White British	19 (79.2)
Asian/Asian British	5 (20.8)
Current employer	
Police force	6 (25)
Local authority/social care	6 (25)
Third sector	3 (12.5)
NHS Trust	3 (12.5)
Academic/Research	6 (25)
Length of time on current role	
1–2 years	5 (20.8)
3–5 years	2 (8.3)
More than 7 years	17 (70.8)

Five key themes and twelve subthemes were identified: (i) risk and protective factors of missing incidents, (ii) gaps in reporting and response pathways, (iii) the use of technology for preventing and responding to missing incidents, (iv) the role of communities in prevention of harm, and (v) the need for integration of services through multi-agency collaboration (Table 2).

**Table 2:** Main themes and subthemes from the interviews and stakeholder forum.

Main themes	Sub-themes
Risk and protective factors of missing incidents	Risk factors Cognitive, and psychological factors Physical health as a risk factor (for both the person with dementia and carer) (carer data only) Environmental risks Limited access to support services Progression of dementia (carer data only) Protective factors Family support Living arrangements
Gaps in reporting and response pathways	Lack of clear protocols and delayed response to missing incidents Limited awareness of resources (carer data only) Fear and uncertainty due to lack of clear pathways (carer data only) Missing incidents not a priority of services (carer data only)
The use of technology for preventing and responding to missing incidents	Challenges in the use of technology Integrating technology in adult social care
The role of communities in prevention of harm	The importance of awareness and support The role of social care and the police
The need for integration of services through multi-agency collaboration	The importance of training and cross-organisational collaboration The need for timely intervention

### Risk and protective factors of missing incidents

#### Cognitive and psychological risk factors

Several cognitive, and mental health conditions were identified as increasing risk of missing incidents. These included the person with dementia experiencing confusion, disorientation, and anxiety symptoms. Many family carers and professionals described situations where people with dementia experienced a missing episode due to reverting to past routines, such as going to work or picking up children from school, which was often associated with leaving their home unexpectedly:

‘They might be reverting back to a time, 15, 20 years ago, when they were going to work, and think, oh, I’ve got to go to work or pick up the kids.’- Healthcare professional.

#### Physical health as a risk factor (for both the person with dementia and carer)

The following themes were identified exclusively from the accounts of family carers: certain physical health conditions, such as infections, dehydration and delirium were also seen as worsening confusion that may contribute to a missing episode. Physical health limitations experienced by carers such as mobility issues were often described as factors that hindered carers from preventing people with dementia from leaving their home or not able to respond to a missing incident in a timely manner.

#### Environmental risks

Living in rural areas was identified as increasing risk of a missing episode. Both carers and professionals identified living in a rural area as contributing to delays in locating someone reported missing, hindering both the time and efficiency of searches. Stressful home environments, walking, or stepping out for some fresh air, were also identified by stakeholders as factors increasing risk of missing episodes:

‘Stepping outside for a brief moment, such as to get fresh air, can unexpectedly result in getting lost.’—Healthcare professional.

#### Limited access to support services

Both carers and professionals highlighted that lack of access to services such as risk assessments resulted in families receiving less support, which could increase vulnerability of experiencing a missing episode:

‘Increased risk occurs when people are on a waiting list for social services and haven’t been assessed yet.’—Healthcare professional.

#### Progression of dementia

As dementia progressed, family carers described situations where high levels of caregiver burden and being overwhelmed with caregiving duties could place people with dementia at risk of experiencing a missing episode; some carers described situations where a missing episode would often trigger placement into long-term care:

‘…after this episode, he needed to be in a care home because it was beyond what we could have done ...’—Family carer.

#### Protective factors: family support and living arrangements

Despite these risks, certain protective factors were also identified that could reduce the occurrence of missing incidents. These were common amongst both carers and professionals and included living with family, having a strong family network, or young members of the family monitoring the person more closely and sharing caring duties with older family carers. Living in a supportive community or neighbourhood, with several people helping when needed, was also seen as a protective factor that could prevent harm associated with a missing incident:

‘A close family network... younger family members are more aware and willing to ask for help when needed, living with family offers protection.’—Police officer.

### Gaps in reporting and response pathways

#### Lack of clear protocols and delayed response to missing incidents

Standardised and clear response pathways for reporting and responding to missing incidents were described as essential by both carers and professionals.

#### Fear and uncertainty due to lack of clear pathways

However, carers additionally described situations where lack of clear care pathways often resulted in fear and uncertainty about the safety and well-being of people with dementia:

‘The struggles of caring for a loved one with dementia are overlooked. When my mum wandered, kind strangers kept her safe, but authorities gave little priority.’—Family carer.

#### Missing incidents not a priority for services

Analyses of carer data showed that this was often related to feeling concerned about whether missing incidents experienced by people with dementia were dealt with as an emergency by local authorities or the police. Some families experienced dismissal when seeking help, with a poor response from authorities, or delayed action:

‘I think it was wrong of the police to say that she wasn’t a priority. If she wandered into the woods, she could have been attacked...’—Family carer.

Both carers and professionals placed particular emphasis on the lack of clear guidelines on what families should do in the event of a missing episode. This was perceived as resulting in confusion for both families, and professionals, as well as members of the public who often assist in missing incident investigations:

‘I don’t think there’s a clearly defined route, in terms of when somebody’s missing.’—Police officer.

Inconsistent approaches from local authorities were described as a key barrier for both groups. The lack of clear support systems in place for missing incidents often resulted in severe distress for families, especially when the person went repeatedly missing. Several stakeholders described how the lack of emergency services available outside of regular hours could potentially contribute to exposure to severe harm for people with dementia. National initiatives to standardise responses to missing incidents were perceived as services that were urgently needed.

#### Limited awareness of resources

Carer data showed that many family carers described being unaware of existing resources, such as the Herbert protocol (a form completed by families or carers providing information about the person with dementia that assists the police in locating them), and how this could be accessed to locate a person missing or safeguard the person from harm. Delays in responses to missing incidents as a result of limited resources available raised concerns for both families and professionals that the person with dementia could experience harm:

‘My worry is that they’re lost somewhere, or being in a waiting list or something more serious happens.’—Healthcare professional.

### The use of technology for preventing and responding to missing incidents

#### Challenges in using technology

Despite the potential benefits, several challenges were identified when implementing technology to prevent or respond to missing incidents. One of the key issues identified by both groups was digital exclusion, with many people with dementia and family carers unable to access digital tools, such as smartphones, GPS trackers or other devices that could assist in locating a person going missing:

‘…for the majority of things, like apps, by their nature, are inaccessible because they rely on smartphones, which not everybody would have.’*—*Family carer.

#### Integrating technology in adult social care

Complex high-tech applications were also seen as creating barriers for both carers and healthcare professionals. Although tracking devices were seen as useful, these were described as limited in locating people with dementia who go missing especially in long distances. Many stakeholders (carers and professionals) described the importance of integrating technology with adult social care services. Most participants agreed that tracking devices can be helpful but should be mainly used as a preventative measure rather than as a dementia care intervention:

‘Adult social care should be funding these devices because they help keep people in their own homes, which is where they want to be, rather than moving them into care homes.’—Stakeholder forum.

Ethical considerations and the need for a clear and accessible information package to ensure widespread understanding and adoption of devices were also described as key by both groups:

‘I think some people are just quite fearful of approaching all of that. So, a really coherent package would be useful, as well as thinking through how that gets to people.’—Stakeholder forum.

### The role of communities in prevention of harm

#### The importance of awareness and support

Community engagement was essential in preventing missing incidents and supporting people with dementia and their families live safe in their own home. Informal support networks and enhanced public awareness were described as important public health mechanisms of keeping people with dementia safe in the event of a missing episode:

‘My person’s away from home. I don’t think it needs police, but I would like everyone in this town to keep an eye out for them without it having to escalate.’—Family carer.

#### The role of social care and of the police

While police-led initiatives provide immediate assistance when someone goes missing, most participants agreed that long-term support should be provided by social care and health services. Interventions in these settings should also be sustainable and easily accessible. Enhancing awareness of professionals, carers and members of the public was perceived as an important initiative that would safeguard people with dementia long-term:

‘The public are encouraged to look at dementia, but not what to do if they identify someone is obviously confused, vulnerable, and maybe out of place. Because I do think that we need to rely on the community as part of the answer to this.’—Stakeholder forum.

### The need for integration of services through multi-agency collaboration

#### The importance of training and cross-organisational collaboration

Lack of dementia training amongst professionals often resulted in miscommunication and delays in supporting families. Although specific tools such as the Herbert Protocol were seen as crucial in aiding police searches, awareness remained inconsistent. One suggestion was the need for a specialist officer to ‘cascade’ training to other team members, ensuring knowledge is effectively shared. Many stakeholders (carers and professionals) expressed concerns over staffing shortages and the challenges for dedicated services for missing incidents in dementia:

‘The insufficient staffing and lack of trained personnel to respond early, as well as the frequent turnover of staff in adult social care is a problem.’—Healthcare professional.

A coordinated approach across partner organisations, such as national health services, local authorities and police services, was perceived as key in prevention and response:

‘Vital that partner organisations are aware of initiatives... they need to be telling people this is available and encouraging people to sign up.’—Stakeholder forum.

While police were seen as better equipped for urgent interventions associated with missing incidents, assessments of risk were described as actions by health and social care professionals, especially for those experiencing repeat missing episodes. Integrated services and multi-agency collaboration were perceived by both carers and professionals as mechanisms that are more likely to result in efficient prevention and management of missing incidents:

‘It’s vital that partner organisations are aware of what’s going on. From a dementia perspective, their role is often reactive, whereas NHS and social care take a more preventative approach.’—Healthcare professional.

#### The need for timely intervention

Although tracking devices were seen as a useful preventative intervention, many stakeholders (both carers and professionals) raised concerns about the cost of such devices and the lack of evidence that they are effective in preventing harm. Several police officers highlighted the need for follow-up assessments for all families that have experienced a missing episode. Psychoeducation for families, and risk assessments were described as key components of future care plans that should be in place ideally at time of diagnosis.

## Discussion

This study is the first to examine the lived experiences of missing incidents in dementia from the perspectives of family carers, identifying several novel risk factors that may contribute to such events. It further explored how both carers and professionals perceive pathways to improve care, revealing systemic challenges in the way societies, health care systems, and statutory services respond to missing incidents in dementia [7]. The results highlight the lack of coordinated approaches to safeguarding people with dementia who go missing, an issue that remains critically important for families, social care services, and law enforcement [23]. Such fragmentation reflects broader patterns in dementia care, including gaps in timely diagnosis, needs assessment and end-of-life care [24].

Findings indicate that fragmented service coordination, poor inter-organisational communication, and missed opportunities for early intervention constitute significant barriers to effective care and the prevention of harm [7, 25]. A key strength of this study is that it enables actionable recommendations tailored to specific stakeholders. For families and carers, this includes strengthening community networks and providing psychoeducational support to facilitate timely interventions. For social care and healthcare providers, recommendations emphasise the development of clear reporting pathways, systematic risk assessments, and structured follow-up support. For police and first responders, the findings highlight the importance of enhancing multi-agency collaboration and communication, alongside targeted training to better understand the lived experiences of families [26].

While some risk factors, such as cognitive decline and physical health, are difficult to modify, others—such as lack of support or preventative interventions—could be addressed if implemented in a timely manner to reduce the likelihood of serious harm [3, 27, 28]. Family involvement and strong community networks emerged as protective factors, highlighting the importance of coordinated action across households, communities and professional services [7, 29].

Standardising reporting and response pathways, and enhancing multi-agency collaboration were identified as essential steps, but data indicated that these solutions must be considered in the context of resource constraints and digital accessibility. Stakeholders suggested integrating GPS and tracking devices into adult social care [23, 30]; however, consistent with limited evidence of their effectiveness [31], these tools were primarily described as preventative aids. Future research examining effectiveness should also investigate affordability, as well as digital inclusion when implementing such technologies [7]. Amongst other recommendations, public awareness initiatives and professional training were identified as key future aims, perceived by stakeholders as essential for establishing a proactive, effective and sustainable system to prevent and respond to missing incidents in dementia [7, 10].

Overall, our findings highlight the importance of a collective, multi-organisational approach that integrates family and community support with law enforcement, social care and healthcare services. Tailored interventions must recognise the distinct roles and responsibilities of each stakeholder: families provide daily support and monitoring, communities offer social networks and local resources, social care and healthcare providers coordinate care and conduct risk assessments; and the police respond to incidents as first responders.

### Strengths and limitations

A key strength of this study is the inclusion of family carers and professionals, who provided valuable insights into the challenges, risk factors and gaps in existing support systems. Incorporating lived experiences ensures that the research findings are both practical and applicable. Engaging key stakeholders such as family carers, police officers and healthcare professionals, is likely to result in the development of interventions that are more likely to be feasible and easy to implement [26]. Despite these strengths, however, our study has several limitations. The sample size was small and lacked diversity, which may limit the range of perspectives captured. Participation was also self-selecting, meaning that carers with particularly strong experiences, either positive or negative, may have been more likely to take part. In addition, the study was conducted within specific community contexts, which may reduce the transferability of findings to other areas or care systems. While we sought to mitigate bias in data interpretation through independent coding, regular team discussions and consensus-building, the potential for researcher bias cannot be fully eliminated. Despite involving six members of our stakeholder group from local authorities and social care, the study had relatively limited input from the broader social care sector, which may have constrained insights from this perspective.

## Conclusion

Preventing harm associated with missing incidents in dementia requires a comprehensive, multi-sector approach that integrates family psychoeducation, law enforcement services and healthcare support as an integrated service. While several risk factors may not be modifiable, addressing systemic challenges in the provision of dementia care, could potentially reduce harm and distress associated with missing incidents. Standardising reporting pathways, improving inter-agency collaboration, and increasing public awareness are critical steps towards an effective public health response. By strengthening collaboration across social care, healthcare and law enforcement, care for missing incidents can become more proactive, sustainable, and adaptable to the diverse needs of people with dementia and their families.

## Data Availability

The full data can be requested by the corresponding author (v.orgeta@ucl.ac.uk).
